# Expression Patterns of Cancer-Testis Antigens in Human Embryonic Stem Cells and Their Cell Derivatives Indicate Lineage Tracks

**DOI:** 10.4061/2011/795239

**Published:** 2011-07-18

**Authors:** Nadya Lifantseva, Anna Koltsova, Tatyana Krylova, Tatyana Yakovleva, Galina Poljanskaya, Olga Gordeeva

**Affiliations:** ^1^Kol'tsov Institute of Developmental Biology, Russian Academy of Sciences, 26 Vavilov Street, Moscow 119334, Russia; ^2^Institute of Cytology, Russian Academy of Sciences, 4 Tikhoretsky Avenue, Saint Petersburg 194064, Russia

## Abstract

Pluripotent stem cells can differentiate into various lineages but undergo genetic and epigenetic changes during long-term cultivation and, therefore, require regular monitoring. The expression patterns of cancer-testis antigens (CTAs) MAGE-A2, -A3, -A4, -A6, -A8, -B2, and GAGE were examined in undifferentiated human embryonic stem (hES) cells, their differentiated derivatives, teratocarcinoma (hEC) cells, and cancer cell lines of neuroectodermal and mesodermal origin. Undifferentiated hES cells and embryoid body cells expressed MAGE-A3, -A6, -A4, -A8, and GAGEs while later differentiated derivatives expressed only MAGE-A8 or MAGE-A4. Likewise, mouse pluripotent stem cells also express CTAs of Magea but not Mageb family. Despite similarity of the hES and hEC cell expression patterns, MAGE-A2 and MAGE-B2 were detected only in hEC cells but not in hES cells. Moreover, our analysis has shown that CTAs are aberrantly expressed in cancer cell lines and display low tissue specificity. The identification of CTA expression patterns in pluripotent stem cells and their derivatives may be useful for isolation of abnormally CTA-expressing cells to improve the safety of stem-cell based therapy.

## 1. Introduction

Two approaches for pluripotent stem cell line production have been developed. The traditional way consists in the isolation of pluripotent cells from preimplantation embryos or the conversion of embryonic germ line cells into pluripotent stem cells [1–4]. Another approach is experimental genome reprogramming of somatic cells to change their differentiation potential. There are three technologies of reprogramming: somatic cell nuclear transfer, fusion of pluripotent and somatic cells, and induction of pluripotency in somatic cells by introduction of pluripotency-related genes or proteins [5–7]. Despite different origin, all pluripotent stem cell lines display considerable similarity of the basic biological properties: high self-renewal rate and ability of in vitro and in vivo differentiation into a wide variety of cell types. On the other hand, comparative analysis of numerous derived human embryonic stem (hES) cell lines demonstrated that they differed in cell growth rate, gene expression profiles, gene methylation profiles, and microRNA profiles [8–10]. These differences may be due to the genetic background of pluripotent embryonic cells initiating hES cell lines, different culture systems used for their maintenance as well as stochastic events during long-term in vitro cultivation which lead to genomic alterations [11–14]. Moreover, induced pluripotent stem (iPS) cells derived from different somatic cells differ in their differentiation and tumorigenic potentials [15–17]. The variation of transcriptional and gene methylation profiles of human ES and iPS cell lines has been widely discussed [18–24]. 

Numerous studies have shown that long-term cultivation leads to the accumulation of different genetic aberrations and abnormal epigenetic changes by the pluripotent stem cells, and such changes can contribute to genomic instability, cell transformation, and cancer development [11, 13, 25, 26]. Furthermore, most iPS cell lines were generated by overactivation of cell oncogenes C-Myc and Klf4 that might enhance their spontaneous uncontrolled expression in undifferentiated pluripotent stem cells and in differentiating progenitor cells, and, therefore, these cells may be transformed to cancer stem cells [27]. Taken together, all these data indicate that utilized pluripotent stem cell lines require regular monitoring of genetic and epigenetic integrity. In addition, large-scale searching of new gene markers is required for identification of the cells that underwent tumorigenic transformation. 

Cancer-testis-associated antigens (CTAs) may be considered as potential gene candidates specific for transformed cells because they are frequently expressed in different types of cancers but have very restricted expression patterns in normal tissues [28]. All CTAs have been shown to be expressed in male gonads, some of them are expressed in the trophoblast, placenta, and developing central nervous system [29–33]. Several gene families are ubiquitously expressed in somatic and germ cells as well as in cancer cells [30]. In addition, certain CTAs were detected in the mesenchymal stem cells and differentiated hES cells [29, 34]. However, cell functions of most CTA families which include more than 100 genes remain enigmatic. Recent studies have demonstrated that CTAs are involved in the regulation of transcription [35], cell cycle and proliferation [36–39], apoptosis [40], and susceptibility to cytokines in cancer cells [41]. Moreover, the expression of some CTAs including MAGEA, SSX, and NY-ESO families is regulated epigenetically by promoter methylation and histone acetylation mechanisms [42–44]. A subset of CTA proteins has been found to elicit spontaneous humoral and cytotoxic T-cell-mediated immune responses in cancer patients, and, therefore, these antigens could be potential cancer vaccine targets [28, 45, 46].

On the other hand, CTAs may be involved in the specification of the early embryonic lineages. MAGEA and GAGE families have been demonstrated to be expressed specifically in the human germ line during the development of male and female reproductive systems [47–50]. In order to clarify the possible role of CTAs in lineage determination during early development and specificity of CTA expression during pathological tissue development, we have examined CTA expression patterns of MAGE A, B, D, and GAGE families in the pluripotent stem cells, their spontaneously differentiated cell derivatives, and cancer cell lines derived from tissues of neuroectodermal and mesodermal origin.

## 2. Materials and Methods

### 2.1. Cell Lines

We utilized hES cell lines SC5, SC7, and SC3a derived from blastocysts (Institute of Cytology, Russian Academy of Sciences, St. Petersburg) [51]. Mouse ES cell R1 line was kindly provided by A. Nagy (Mount Sinai Hospital, Toronto, Canada), mouse embryonic germ cell (EG) line EGC-10 derived from E10.5 embryos was provided by A. McLaren (WTCR Institute of Cancer and Developmental Biology, Cambridge, UK). All cancer cell lines were obtained from Russian Cell Culture Collection (http://www.rccc.cytspb.rssi.ru/). The following groups of cancer cell lines were used: (1) human teratocarcinoma PA-1 and mouse teratocarcinoma F9, (2) human neuroblastomas IMR-32 and SK-N-MC, (3) human glioblastomas A-172, GL-6 (U-251MG), T-98G, (4) human rhabdomyosarcoma A-204 v and embryonal rhabdomyosarcoma RD, and (5) human osteosarcoma HOS (TE85, clone F5), MG-63, U-2 OS.

### 2.2. Human Tissue Specimens

The human tissue samples of testes and brains were obtained from adult males during postmortem examination in Forensic Medical Examination Bureau, Moscow Department of Public Health. The procedures of human tissue sampling were carried out according to the Funeral Law of Russian Federation and were approved by the Institutional Ethics Committee. After autopsy, the tissue samples were immediately transferred to TRIzol Reagent (Invitrogen).

### 2.3. Mouse Embryos and Tissues Sampling

C57Bl/6 mice at the age of 2-3 months were obtained from the Animal Breeding Facility-Branch “Pushchino” (Institute of Bioorganic Chemistry, Russian Academy of Sciences). Animal keeping and experiments were approved by the Institutional Ethics Committee. Total RNAs were extracted from E 7.5 mouse embryos with removed placenta, fetal gonads associated with attached mesonephros of E11.5 embryos, and male fetal gonads from E14.5 embryos. In addition, total RNAs were isolated from testicle and brain tissue samples of adult mouse males.

### 2.4. Cell Line Maintenance

Human ES cell lines were maintained on human embryonic fibroblast feeder cells (Russian Cell Culture Collection of Vertebrates at the Institute of Cytology) inactivated by mitomycin C treatment (10 ug/mL, Sigma). ES cells and their differentiated cell derivatives were cultivated in KnockOut Dulbecco's modified Eagle's medium (KDMEM) supplemented with 2 mM L-glutamine, 1% nonessential amino acids, 10 ng/mL basic fibroblast growth factor bFGF (Invitrogen), 0.1 mM *β*-mercaptoethanol (Sigma), and 15% Knockout Serum Replacement (Gibco/Invitrogen). Colonies of undifferentiated hES cells were manually detached from the feeder cells, divided into cell clusters and transferred into low adhesion culture plates for spheroid generation or into new plates with feeder cells for following expanding. After isolation from the feeder cells, the hES cell clusters formed embryoid bodies (EBs) within one day of cultivation into low adhesion plates (Greiner Bio-one). At day 10 of cultivation in nonadherent culture, EBs were collected and used for RT-PCR analysis. 

The early cell derivatives of hES SC5 or SC7 cells have been isolated from spontaneously differentiated adherent ES cell cultures maintained without feeder cells for 10–14 days in the hES cell media. Uniform cell outgrowths of extraembryonic endoderm, early neuroectodermal-like rosette structures and mesenchymal-like cells were isolated manually, propagated under feeder-free conditions, and collected for RT-PCR and immunohistochemical analyses. 

Mouse ES and EG cells were maintained on mouse embryonal fibroblast feeder cells inactivated by mitomycin C or in a feeder-free system in media containing leukemia inhibitory factor (LIF, 10 ng/mL). Mouse ES and EG cells, mouse teratocarcinoma F9 and human teratocarcinoma PA-1 cells were cultivated in DMEM supplemented with 2 mM L-glutamine, 1% nonessential amino acids, (HyClone), 0.1 mM *β*-mercaptoethanol (Sigma), and 15% Characterized Fetal Bovine Serum (HyClone). For EB generation R1, EGC-10, F9, and PA-1 cells were placed in “hanging drops” (500 cells per drop) for 3 days. After formation, EBs were collected and cultured for 10 days in low adhesion plates. 

Neuroblastoma, glioblastoma, rhabdomyosarcoma, and osteosarcoma cell lines were maintained as recommended for these cell lines previously (see http://www.rccc.cytspb.rssi.ru/).

### 2.5. Teratoma and Teratocarcinoma Assay

For teratoma and teratocarcinoma to be obtained, we used as recipients immunodeficient nude mice from the Animal Breeding Facility-Branch “Pushchino”. All animal study protocols were approved by the Institutional Bioethical Committee. Mouse ES, EG, and EC cells and human ES and EC cells were injected subcutaneously into nude mice (10^6^ cells per mouse). After tumor development, the animals were sacrificed, teratomas and teratocarcinomas were isolated and fixed by 10% paraformaldehyde (Sigma), dehydrated according to the standard method, and embedded into paraffin for sectioning. Histological preparations were stained by hematoxylin and eosin and examined under a Leica DMRXA2 microscope.

**Karyotyping**

To prepare metaphase chromosomes, 0.1 *μ*g/mL colcemid (Karyomax, Gibco, USA) was added to the culture media 4 h prior to cell fixation. After dissociation, the cells were treated with hypotonic solution of 0.075 M KCl and 1% sodium citrate and then fixed by glacial acetic acid (3 : 1). Metaphase spreads were prepared by dropping cells onto glass slides followed by air drying. Preparations of metaphase spreads were stained by Giemsa solution. For the karyotype analysis, routine G-banded technique were applied. No less than 30 metaphase spreads for SC5, SC7, and SC3a were analysed. The hES cell karyotypes were studied by an Axio Imager M1 microscope (Carl Zeiss, Germany) with Ikaros4 Karyotyping System (MetaSystems, Germany) and described according to the International System for Human Cytogenetic Nomenclature.

### 2.6. Detection of Alkaline Phosphatase Activity (ALP) and Immunostaining

 Human and mouse ES, EG, and EC cells and EBs were fixed by 2% paraformaldehyde in phosphate-buffered solution, PBS, pH 7.0 within 15 min. ALP activity was detected after incubation in a solution containing 10 mL 0,02 M Tris-HCl buffer (pH 8,7), 1 mg Naphtol-AS-B1-phosphate, and 5 mg Fast Red dye Texas Red (all from Sigma) at 37°C for 1 h. 

For immunofluorescence analysis, cells fixed in 4% paraformaldehyde in PBS for 1 h were washed and permeabilized with 0.5% Triton X-100 (Sigma). Nonspecific reaction was blocked by 10% chicken serum (Gibco/Invitrogen). Cells were incubated in a solution of primary antibodies in PBS-Tween 20 at 4°C overnight. Primary antibodies rabbit anti-Oct4, goat anti-GATA4 (Santa Cruz Biotechnology), goat anti-Nanog (R&D Systems), mouse anti-Nestin (Abcam), anti-*α*-Actinin (Sigma), and rabbit anti-BRY (Abcam) were used in dilution 1 : 100. Secondary chicken antirabbit, donkey antigoat, and chicken antimouse antibodies conjugated with Alexa Fluor 594 and Alexa Fluor 488 (Molecular Probes) were diluted at 1 : 900 in blocking buffer and applied to cells for 3 h at room temperature. DAPI (Molecular Probes) was applied for nuclear staining for 20 min. Cells were mounted and examined under a Leica DMRXA2 fluorescent microscope. For negative controls, primary antibodies were omitted and the same staining procedure was used.

### 2.7. RNA Isolation and RT-PCR Analysis

Total RNAs were extracted from all cell lines, human and mouse tissues, and mouse embryonic samples using TRIzol Reagent (Invitrogen) according to the manufacturer recommendations. Each sample was treated with TURBO DNase (Ambion/Invitrogen) to avoid DNA contamination, and 1 *μ*g of total RNA from each sample was reverse transcribed using RevertAid M-MuLV revertase and random hexamer oligonucleotide primers (Fermentas) for cDNA synthesis. PCR reaction mixtures were prepared according to the manufacturer protocol for Taq polymerase (Silex). Probes were denaturated at 94°C for 5 min and cycled at 94°C for 45 s, at 58°C for 45 s, and at 72°C for 45 s followed by final extension at 72°C for 5 min after the completion of 30 cycles. The expression of housekeeping genes (human RPL19 and mouse Hprt genes) was used for the normalization of PCR reaction. Primer sequences and size of their expected products are represented in Tables S1 and S2 (see Supplementary Material available online at doi: 10.4061/2011/745239). Primer pair for GAGE1, 2, 10, 12, 13 was borrowed from [29].

## 3. Results

### 3.1. Expression of CTA Genes in Adult Human and Mouse Testicular and Brain Tissues

**Figure 1:**
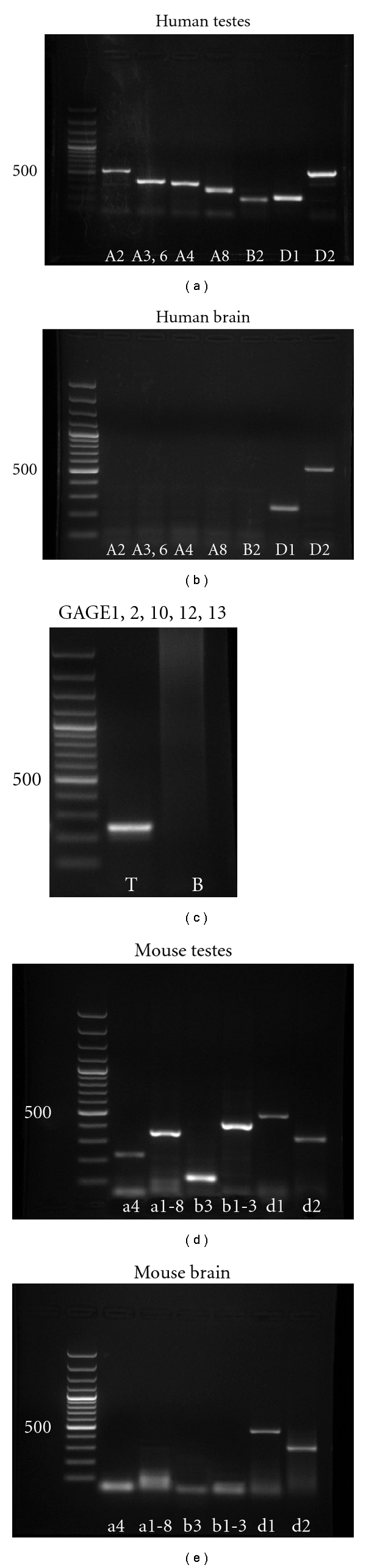
CTA expression in adult human and mouse testicular and brain samples. Designations: T: human testes, B: human brain.

At first, we tested the primers for the detection of CTA expression by RT-PCR in normal human and mouse tissues used as positive and negative controls. The expression of MAGE-A2, -A3, -A6, -A4, -A8, MAGE-B2, MAGE-D1, -D2, and several members of the GAGE family (GAGE-1, -2, -10, -12, -13) was detected in human testes whereas only MAGE-D1 and MAGE-D2 were expressed in human brain samples (Figures 1(a)–1(c)). Likewise, we detected the expression of Mage-a4, Mage-a 1, 2, 3, 5, 6, 8, Mage-b 1-3, Mage-b3, Mage-d1, -d2 in mouse testes and Mage-d1, -d2 in brains correspondingly (Figures 1(d) and 1(e)). Our analysis has demonstrated that all primers detected only PCR sequences of expected size and did not detect additional nonspecific sequences. In addition, the expression of all CTAs studied was detected only in the testes whereas in normal somatic tissue (brain samples) only MAGE-D1, -D2/Mage-d1, -d2 have expressed as expected.

### 3.2. CTA Gene Expression in Human ES and EC Cells

**Figure 2:**
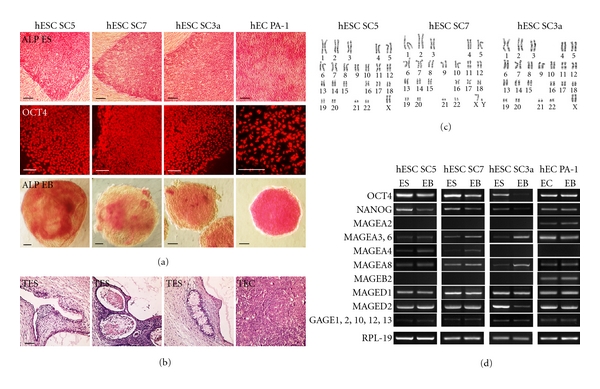
CTA expression patterns in hES SC5, SC7, and SC3a cells and hEC PA-1 cells. (a) Activity of alkaline phosphatase and OCT4 in undifferentiated hES and hEC cells and in EBs formed by these cell lines. Scale bar = 100 *μ*m. (b) Histological sections through teratomas and teratocarcinomas formed by hES and hEC cell lines. Cell derivatives of three germ layers were found in teratomas (TES) formed by hES cells and entirely cancer cells in teratocarcinomas (TEC). Scale bar = 100 *μ*m. (c) Normal diploid karyotypes of hES SC5, SC7, and SC3a cells: SC5—46, XX; SC7—46, XY; SC3a—46, XX. (d) Expression of CTAs in hES SC5, SC7, and SC3a cells, hEC PA-1 cells and EBs formed by these cells.

Human ES cell lines SC5, SC7, and SC3a were recently derived and characterized as pluripotent stem cells by standard in vitro and in vivo assays [51] and (Figures 2(a)–2(c)). The cytogenetic analysis of these cell lines has shown that they retained normal diploid karyotypes during at least 30 passages: 46, XX for SC5 and SC3a, and 46, XY for SC7 (Figure 2(c)). However, the differentiation potential of these lines has been found to be different. Human ES cell lines SC5 and SC7 formed teratomas with derivatives of three germ layers (Figure 2(b)) while SC3a cells were more prone to differentiation in vitro and had restricted capacity to grow in teratomas. 

In our experiments, undifferentiated SC5, SC7, and SC3a cells and EB cells expressed key pluripotency genes OCT4 and NANOG and displayed a high ALP activity (Figures 2(a) and 2(d)). In differentiating 10-days EBs, the expression of OCT4 and NANOG was downregulated, and ALP activity was diminished (Figures 2(a) and 2(d)). Analysis of CTA expression has shown that in undifferentiated hES cell lines SC5, SC7, and SC3a only MAGE-D1, -D2 were expressed at a high level while other CTA genes were either not expressed or expressed very weakly (Figure 2(d)). It is plausible that the low level of expression could be due to few differentiated cells which sometimes contaminate undifferentiated cell cultures. On the other hand, these CTAs may be expressed in both undifferentiated and early differentiated hES cells. In two hES cell lines, SC5, and SC7, the expression of MAGE-A3, -A6, -A4, -A8 and GAGEs was detected in undifferentiated hES cells and in 10-day EBs formed by these lines. However, the undifferentiated hES cells and EBs of SC3a line expressed MAGE-A3, -A6, -A8, and GAGEs but did not express MAGE-A4. The expression of MAGE-A2 and MAGE-B2 was not detected in any lines studied. In addition, the expression profile of each hES cell line remained stable during passages (data not shown). 

Nullipotent teratocarcinoma PA-1 cells are the malignant counterpart of pluripotent stem cells that lost completely the ability of differentiation (Figures 2(a) and 2(b)). EC PA-1 cells and EBs have expressed high level of OCT4 and NANOG and almost all CTAs studied, MAGE-A2, -A3, -A6, -A8, MAGE-B2, MAGE-D1, -D2, and GAGE family genes. Interestingly, like hES SC3a cells, PA-1 cells did not express MAGE-A4 (Figure 2(d)). Our analysis of hEC PA-1 cells and hES cells has shown that their CTA expression profiles were very similar, but, at the same time, they differed in the expression of two CTAs.

### 3.3. CTA Gene Expression in Differentiated Human ES Cell Derivatives

**Figure 3:**
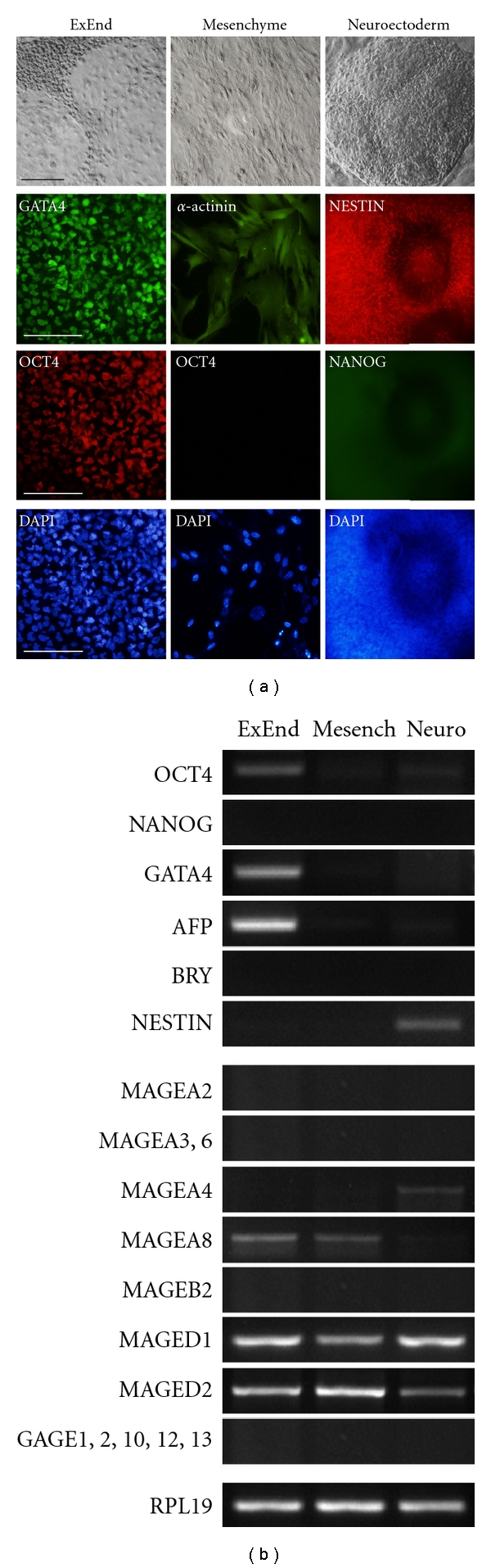
Expression of CTAs and lineage-specific genes in differentiated hES cell derivatives. (a) Morphology and immunostaining of extraembryonic endoderm derivatives by antibodies against GATA4 and OCT4, mesenchymal cells against *α*-ACTININ and OCT4, neuroectodermal cells against NESTIN and NANOG. Scale bar = 100 *μ*m. (b) Expression profiles of CTAs and lineage marker genes in hES cell derivatives.

We studied three types of early differentiated cell derivatives of human ES SC5 and SC7 cells that predominated during spontaneous hES cell differentiation in vitro. These cells were easily distinguished from other cells in morphology and could be easily separated from other cells in cell outgrowths (Figure 3(a)). To identify cell types, we analyzed specific gene and protein expression in the studied samples. The expression of specific marker genes for pluripotent (OCT4, NANOG), extraembryonic endoderm (GATA4, AFP), neuroectodermal (NESTIN) and mesenchymal-like (BRY and *α*-Actinin) cells was tested by RT-PCR (Figure 3(b)) and immunohistochemical staining (Figure 3(a)). Analysis of CTA expression in these selected cell derivatives has shown that the extraembryonic endoderm cells expressing high levels of GATA4 and AFP and low level of OCT4 express MAGE-A8 and MAGE-D1, -D2 as well (Figure 3). Mesenchymal-like cells that were *α*-Actinin positive but BRY-negative had CTA expression profile similar to that of extraembryonic endoderm cells (Figure 3). In contrast, the neuroectodermal cell derivatives expressed NESTIN and MAGE-A4 (Figure 3). Moreover, all three cell types expressed MAGE-D1, -D2 genes and did not express GAGE genes.

### 3.4. Expression of CTAs in Human Cancer Cell Lines of Neuroectodermal and Mesodermal Origin

**Figure 4:**
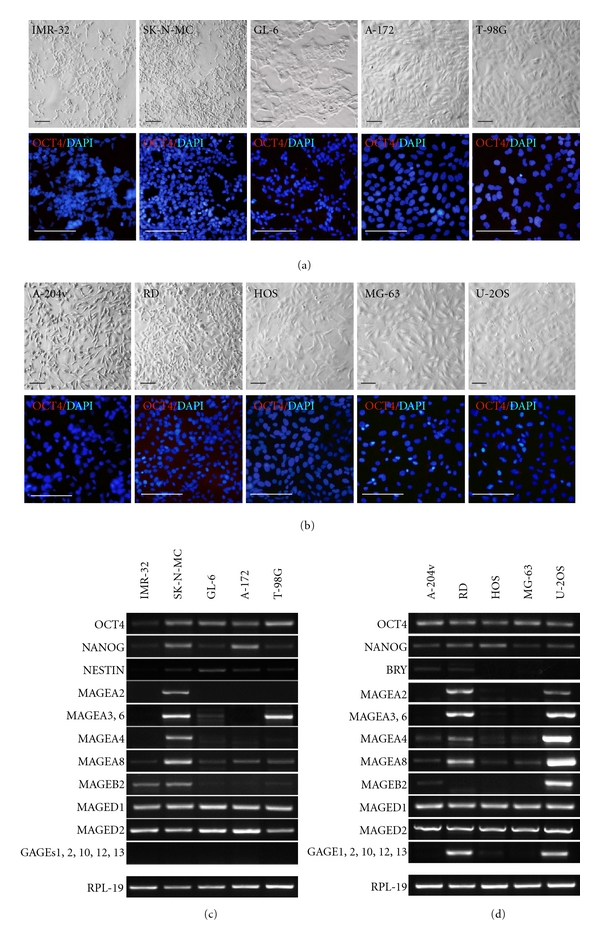
CTAs in cancer cell lines derived from tissues of neuroectodermal and mesodermal origin. (a) Cell morphology of neuroblastomas and glioblastomas and immunostaining of these cell lines by antibody against OCT4. Scale bar = 100 *μ*m. (b) Immunocytochemical analysis of OCT4 expression in rhabdomyosarcoma and osteosarcoma cell lines. Scale bar equal 100 *μ*m. (c) CTA expression profiles of cancer cell lines of neuroectodermal and mesodermal lineages.

In order to determine whether CTA-specific expression patterns were associated with histological origin or with other characteristics of tumors, we tested cell lines which were derived from the embryonic (neuroblastoma, embryonal rhabdomyosarcoma), childhood (rhabdomyosarcoma, osteosarcoma), and adult (glioblastoma) cancers of mesodermal and neuroectodermal origin. In addition, the expression of OCT4 and NANOG was studied in all cancer cell lines (Figures 4(a) and 4(b)).

In the neuroblastoma cell lines IMR-32 and SK-N-MC, CTA expression patterns were markedly different. SK-N-MC cells expressed all MAGEs tested while IMR-32 cells only two (Figure 4(c)). However, the expression of MAGE-B2 and different levels of MAGE-A8 were detected in both cell lines. All three glioblastoma cell lines, GL-6, A-172, T-98G, expressed MAGE-A8 and very weak level of MAGE-B2. Two of three cell lines, GL-6 and T-98G, expressed MAGE-A3. Thus, most cancer cell lines from tissues of neuroectodermal origin expressed MAGE-A8 and MAGE-B2 mRNAs but embryonic tumors (neuroblastomas) expressed significantly higher level of MAGE-B2. The expression of GAGEs was not detected in any of neuroectodermal cancer cell lines (Figure 4(c)).

Analysis of CTA expression profiles of cancer cell lines from the tissues of mesodermal origin has shown that all of them have expressed variable levels of MAGE-A8 and MAGE-A4. Two lines, embryonal rhabdomyosarcoma RD and osteosarcoma U-2 OS, had very similar CTA profiles, except MAGEB 2, and expressed almost all CTAs studied including GAGEs (Figure 4(d)). 

As mentioned above, the expression of OCT4, NANOG, NESTIN, and BRY was tested in cancer cell lines also. Interestingly, mRNAs of OCT4 and NANOG were detected by RT-PCR in all cancer lines while the proteins were not revealed by immunostaining. Likewise, low expression of BRY has been found at mRNA levels in rhabdomyosarcomas, and NESTIN in neuroblastomas and glioblastomas but not at the protein level (Figure 4, and data not shown). We suggest that OCT4 and NANOG transcripts detected in all cancer lines studied may be relevant to the pseudogenes and have no functional meaning. Similarly, the expression of BRY and NESTIN at mRNA level may be activated aberrantly in these cells as often observed in cancer cells.

### 3.5. CTA Gene Expression Profiles in Mouse ES, EG, and EC Cells and in Mouse Germ Line Cells

**Figure 5:**
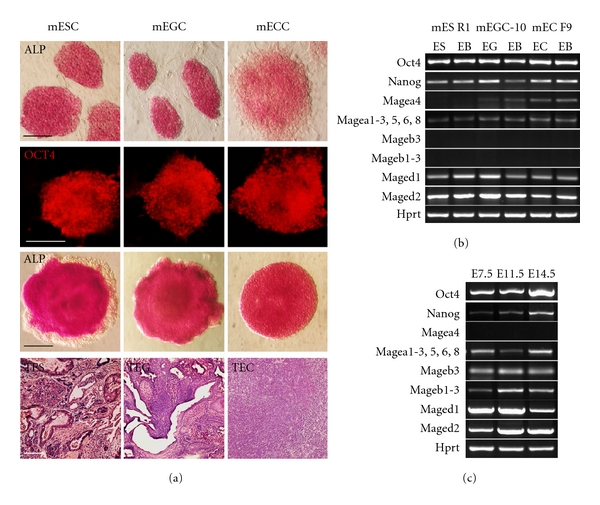
Gene expression profiles of CTAs in mouse ES, EG, and EC cells and in mouse germ line cells. (a) Pluripotent ES and EG cells and nullipotent EC cells express alkaline phosphatase and Oct4 and develop EBs; ES and EG cells differentiate into derivatives of three germ layers in teratomas (TES, TEG) while EC cells grow in vivo as malignant tumors (TEC). Scale bar equal 100 *μ*m. (b) Expression of CTAs, Oct4, and Nanog in mouse ES, EG, and EC cells and EBs. (c) CTA expression profiles of germ line cells isolated from embryos of E 7.5, E 11.5 and E 14.5 stages.

The murine Mage genes, like their human homologues, are expressed in a wide variety of tumors, in fetal and adult male gonads, and in several embryonic and extraembryonic tissues [52–57]. In order to determine whether specific expression patterns of Mage-a and Mage-b genes can be attributed to pluripotent stem cell differentiation, CTA expression profiles were examined in mouse pluripotent stem cells (ES and EG cells) and embryonal teratocarcinoma cells (Figures 5(a) and 5(b)). Moreover, we compared them with the profiles of mouse primordial germ cells at the critical stages of germ line development: epiblast cells at early gastrulation stage, postmigratory primordial germ cells just after their occupation of developing genital ridges and gonocytes of male embryonic gonads. Our results suggest that the pluripotent ES and EG cells, nullipotent teratocarcinoma EC cells and their EB cells expressed Mage-a1, 2, 3, 5, 6, 8 and Mage-d1, -d2 but did not express CTAs of Mage-b family. Furthermore, the expression of Magea-4 gene was detected in EGC-10 and EC F9 cells but not in ES R1 cells (Figure 5(b)).

The profiles of embryonic germ line cells at the studied stages were very similar because all CTAs tested, except Mage-a4, were expressed in all types of germ line cells (Figure 5(c)). Interestingly, in contrast to other CTAs, Mage-a 4 was expressed in adult mouse testes but not in the early primordial germ cells and gonocytes. Thus, the CTA expression profiles of mouse ES, EG, and EC cells significantly differed even from those in the early epiblast cells. On the other hand, mouse and human embryonic germ line cells expressed similar CTA families except GAGE antigens which were not identified in mice.

## 4. Discussion

The expression of most CTA genes in the normal tissues is restricted to adult testicular germ cells but is aberrantly activated in various types of cancers. Moreover, CTAs have been found to be expressed in embryonic germ and somatic cells as well as in the extraembryonic structures [29, 50]. We hypothesized that unique expression pattern of CTAs in germ cells may be a part of the developmental program which includes the restriction of germ line from somatic cells during the early gastrulation stages. On the other hand, deviations from the normal lineage specification can be associated with disturbances of the typical CTA expression patterns. We investigated the CTA expression patterns during ES cell differentiation in vitro using them as a model of normal lineage specification and compared CTA profiles of undifferentiated ES cells, their differentiated cell derivatives, and cancer cell lines of neuroectodermal and mesodermal origin in order to identify coincident and discriminating gene subsets. 

Our results suggest that undifferentiated pluripotent hES cells expressed low mRNA levels of several CTAs, MAGE-A3, -A6, -A4, -A8, and GAGEs. Note that MAGE-A4 was not expressed in hES SC3a cell line that differed from SC5 and SC7 cells in their growth and differentiation potential [51]. However, unlike undifferentiated cells, the early hES cell derivatives expressed only one gene of MAGEA family and did not express CTAs of GAGE family. It can not be ruled out that MAGE-A4 and -A8 expression may be referred to both undifferentiated hES cells and extraembryonic endoderm, mesenchyme-like and neuroectoderm cells also residing in EBs whereas MAGE-A3, -A6, and GAGEs were expressed exclusively in pluripotent cells. 

Previously, Gjerstorff et al. [29] also detected MAGE-A2 and MAGE-A6 expression in EBs of KMEB1 and KMEB2 hES cell lines and cell derivatives of these lines in teratomas but not in undifferentiated hES cells. However, only 3 of 6 hES cell lines studied expressed CTAs during in vitro and in vivo differentiation. Moreover, the expression of GAGEs was not detected either in hES cells or in EBs. In our experiments, the same primer pairs were used for GAGE expression analysis but low levels of GAGE 1, 2, 10, 12, 13 transcription were detected in both undifferentiated hES cells and EBs in all cell lines studied. Conversely, we did not observe the expression of MAGE-A2 in any hES cell lines or their differentiated cell derivatives but identified MAGE-A2 mRNA in EC PA-1 cells. As mentioned above, SC5 and SC7 hES cell lines had very similar CTA expression profiles and similar growth and differentiation potentials, and, moreover, each hES cell line has retained its specific CTA profile during long-term cultivation. We suppose that interlinear variations in CTA expression patterns may be characteristic for a certain hES cell line state.

Mouse pluripotent ES and EG cells also expressed several genes of Mage-a family, but, surprisingly, Mage-a4 was expressed only in mouse EG and EC cells. Furthermore, the expression of Magea4 was not detected in mouse primordial germ cells of E 11.5 and E14.5 embryos as well, although Mage-a4 was expressed in adult male gonads. Mouse embryonic and adult germ cells also expressed Mage-b1, 2, 3 genes unlike mouse pluripotent stem cells. Similarly, the expression of MAGE-B2 was found in adult human testes but not detected in human ES cells or their cell derivatives. Thus, the CTA expression profiles of mouse ES, EG, and EC cells significantly differed even from the early epiblast cells as well as from primordial germ cells. Hence, the pluripotent stem cells in vitro may not be quite equivalent to any of these cell types in embryos. Nevertheless, the similarity of CTA expression profiles of human and mouse pluripotent stem cells suggests their similar cell state and origin. 

Little is known about CTA expression during the development of the germ and somatic lineages in mammals. Previous studies have shown that during human primordial germ cell determination, differentiation, and gonad maturation, the CTA expression patterns undergo significant changes. Firstly, GAGE proteins were expressed in the male and female primordial and mature germ cells, from 5–8 weeks of gestation until adulthood [29, 49, 50]. The expression of MAGE-A1, MAGE-A4, and NY-ESO-1 in the fetal testes and ovaries is initiated later than GAGE expression and terminated at different development stages in male and female fetal gonads [47, 48, 50]. MAGE-A4 has been found to be expressed firstly in single cells of the fetal testes at the age of 17 weeks, and, thereafter, the number of MAGE-A4-positive gonocytes and spermatogonia progressively increased until 28 weeks. MAGE-A1 and NY-ESO-1 proteins were also detected in the fetal testes at the age of 9 weeks and in ovaries at the age of 13 weeks but these proteins displayed differential expression patterns through male and female germ cell development. Our data about the Mage-a, Mage-b, and Mage-d expression patterns in the mouse primordial and fetal germ cells also demonstrate for the first time the CTAs implication in an early mouse germ line development as well as similarities and dissimilarities of CTA expression patterns in mouse and human embryonic cells. 

Apart from germ cells, the MAGE-A family members were expressed in somatic lineages, in particular, in human developing central nervous system and peripheral nerves as well as in myotome and myoblasts at the early stages (from 5 to 8 weeks) but no MAGE-A expression was detected in the neural structures of 17- and 23-weeks old fetus or in the adult brain [29]. Moreover, these authors reported that GAGE proteins were expressed in the early ectodermal and neuroectodermal cells but their expression disappeared in the later differentiated cells of these lineages. In addition, MAGE-A protein expression did not correlate with the GAGE expression pattern in the neuroectodermal cells as opposed to germ cells. Our findings that MAGE-A4 and MAGE-A8 are expressed in the early mesenchymal and neuroectodermal cells derived from hES cells are consistent with the previous observations on human fetal tissues and differentiated hES cells in teratomas [29]. On the other hand, we did not find GAGE expression in the above mentioned differentiated cell types. Therefore, further detailed examination of CTA expression pattern in different cell types derived from pluripotent cells during in vitro differentiation and in embryonic tissues can clarify this discrepancy. 

**Figure 6:**
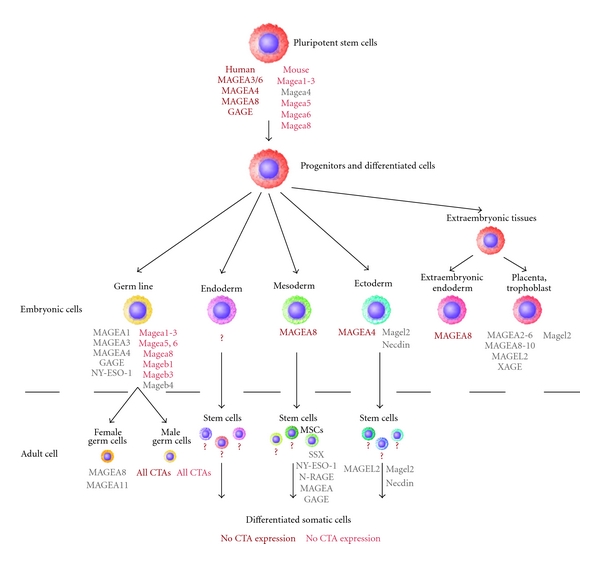
CTAs expression in pluripotent cells and derivatives of different lineages. Data are summarized from present study (marked by brown and red) and [29–31, 34, 47–50, 52–57]. Human CTAs are marked with capital letters.

The expression of CTAs was also detected in extraembryonic tissues. We identified MAGE-A8, MAGE-D1, -D2 expression in extraembryonic endoderm cells derived from hES cells. Previously, in immunohistochemical study of more than 50 human placenta samples of different gestational ages, the high level of MAGE-A3 and MAGE-A4 expression was revealed while the expression of NY-ESO-1 and GAGEs was sporadic [31]. Genome-wide analysis of CTA expression in the normal and cancer tissues displayed the MAGE-A 2–6, 8–11, and XAGE expression in placenta [30]. Human MAGEL2 gene and its mouse homologue Magel2 were detected in human and mouse placenta [56]. Taken together, all these data demonstrate that CTAs are involved in germ line and somatic lineage development of different mammals. Specific spatio-temporal patterns of CTAs expression may be associated with the determination and specification of different lineages both in the embryo and during pluripotent stem cell differentiation (Figure 6). 

Another aspect of our work was the analysis of CTA expression profiles of teratocarcinoma cell lines that are malignant counterparts of pluripotent stem cells and cancer cell lines derived from the tissues of neuroectodermal and mesodermal origin in order to find the specificity of CTA expression during normal and pathological tissue development. Initially, we compared CTA profiles of undifferentiated hES cells, hEC cells, and EBs formed by these cell lines. The hES and hEC cell CTA profiles were very similar, but hEC cells expressed two additional genes, MAGE-A2 and MAGE-B2, which were not expressed in the pluripotent stem cells. Moreover, hEC PA-1 cells did not express MAGE-A4 like hES SC3a cell line. Interestingly, the CTA profiles studied of mouse ES cells differed from those of mouse EC cells only in Mage-a4, and, moreover, mouse EC F9 cells expressed Mage-a4 as opposed to human EC PA-1 cells. Nevertheless, Mage-a4 was also expressed in the normal mouse EG cell line. Thus, MAGE-A4/Mage-a4 expression pattern is variable in murine and human pluripotent and teratocarcinoma cells, and the question of specificity of this expression in pluripotent cells remains open. Recent studies of normal testes and different types of germ cell tumors have shown that normal spermatogonia and seminoma cells specifically expressed MAGE-A4 while anaplastic seminoma and nonseminoma germ cell tumors were negative for this antigen [47]. On the other hand, the expression of MAGE-A2, -A3, -B1, and -B2 was also found in most seminomas studied while MAGE-A2 and MAGE-A4 were expressed in pure embryonal carcinoma tumors, as well as in a half of pure yolk sac tumor samples [33]. In addition, in our study hEC PA-1cells expressed MAGE-A2 and MAGE-B2 that were not detected in hES cells, and, therefore, these CTAs may be considered as gene candidates for their further investigation as early markers of transformed pluripotent cells.

Analysis of CTA expression in the neuroectodermal cancer lines has shown that MAGE-A8, MAGE-B2, and MAGE-A3, -A6 together with MAGE-D1, -D2 are expressed in most cell lines tested whereas the early neuroectodermal cells derived from hES cells expressed only MAGE-A4 and MAGE-D1, 2. Thus, no common genes of MAGE-A, MAGE- B, and GAGE families were revealed in the normal and cancer cells of this lineage. On the other hand, the frequencies of MAGE-A1 and MAGE-A3 expression in different astrocytomas and glioblastomas have been demonstrated to vary in range from 0 to 30% [58–60]. Hence, CTA expression patterns in brain tumors may have low tissue specificity. Also, we determined MAGE-A8 and MAGE-A4 as common CTAs in expression profiles of rhabdomyosarcoma and osteosarcoma lines, and MAGE-A8 was also found in the mesenchymal derivatives of hES cells. Interestingly, the cancer cell lines of both neuroectodermal and mesodermal origin expressed MAGE-A8 while normally it was expressed in the mesenchymal hES cell derivatives only. 

Relatively recently it was found that several CTAs, including SSX, NY-ESO-1, and N-RAGE, were also expressed in undifferentiated mesenchymal stem cells (MSCs) derived from adult bone marrow or embryonic liver, but their expression was downregulated after osteocyte and adipocyte differentiation [33]. These important data require further investigations since it is unclear whether CTA expression is characteristic of MSCs or is activated during their cultivation in vitro likewise in cancers. 

Overall, the frequency of CTA expression is highly variable among different tumor types [28, 30, 60]. For instance, melanoma, ovarian, liver, and lung cancers are “CTA-rich” tumors because they have high frequency of CTA expression while hematopoietic, colon, renal, and pancreas cancers have low frequency of CTA expression. Moreover, the cancers of higher histological grade and later clinical stage as well as metastatic tumors display higher frequency of CTA expression than the primary tumors. The frequency of CTA expression correlates with the worst prognosis. Thus, it is plausible that CTAs are aberrantly activated and expressed in different cancers and have low lineage specificity. However, examination and systematization of CTA expression patterns in embryonic and adult normal cells as well as cancer cells of different types may illuminate whether CTAs are implicated in normal and pathological lineage development.

## 5. Conclusions

We have shown that several CTAs, such as MAGE-A3, -A4, -A6, -A8, and GAGEs, are expressed in the undifferentiated hES cells and early differentiated EB cells while only one gene of MAGE-A family was expressed in the later differentiated cell derivatives of hES cells, MAGE-A8 in the extraembryonic endoderm and mesenchymal cells and MAGE-A4 in the neuroectodermal progenitors. Like hES cells, mouse pluripotent cell lines, ES and EG cells, also expressed CTAs of Mage-a family but did not express Mage-b family genes unlike epiblast and primordial germ cells. Moreover, we detected different expression pattern of MAGE-A4/Magea4 in both human and mouse pluripotent and teratocarcinoma cells. Despite great similarity of CTA expression patterns in hES and their malignant counterparts, hEC cells, two of CTAs studied, MAGE-A2 and MAGE-B2, were detected only in hEC cells but not in hES cells. These CTAs may be considered as marker gene candidates of transformed pluripotent cells for further study. Comparative analysis of CTA profiles of cancer cell lines derived from the tissues of neuroectodermal and mesodermal origin and hES cell-derived progenitor cells of the similar lineages has shown that in most cases CTAs were aberrantly expressed in cancer cells and display low tissue specificity. Thus, further investigation and identification of CTA expression patterns in pluripotent and multipotent stem cells and their derivatives as well as different types of cancers is an important step towards understanding of CTA functions in normal and cancer cells and also may be useful for the isolation and removal of abnormally CTA-expressing cells to improve the safety of stem cell-based therapy.

## Supplementary Material

List of primers for human and mouse genes studied and size of products detected by RT-PCR. The primers were constructed using the data on the structure of the genes studied available from the GenBank, MGI, and Ensemble databases.
