# Supplementary material for: Expression Patterns of Cancer-Testis Antigens in Human Embryonic Stem Cells and Their Cell Derivatives Indicate Lineage Tracks
Source: Stem Cells Int. 2011 Jul 18;2011:795239. doi: 10.4061/2011/795239 (PMC3140037; doi:10.4061/2011/795239)
Supplement: Supplementary file 1 — List of primers for human and mouse genes studied and size of products detected by RT-PCR. The primers were constructed using the data on the structure of the genes studied available from the GenBank, MGI, and Ensemble databases. [file 795239.f1.pdf]

**Table S1:** RT-PCR primers for human genes studied

| Gene | Product (bp) | Primer sequences | Accession no. |
|---|---|---|---|
| OCT4/POU5F1 | 259 | 5'gggtggaggaagctgacaac3' forward | NM_002701 |
| | | 5'gcatagtcgctgcttgatcg3' reverse | NM_203289 |
| NANOG | 368 | 5'tgcctcacacggagactgtc3' forward | NM_024865 |
| | | 5'ctgcgtcacaccattgctattc3' reverse | |
| GATA4 | 352 | 5'tccaaaccagaaaacggaag3' forward | NM_002052 |
| | | 5'aagaccaggctgttccaaga3' reverse | |
| AFP | 312 | 5'gcggcctcttccagaaacta3' forward | NM_001134 |
| | | 5'tttcatccaccaccaagctg3' reverse | |
| BRY | 353 | 5'agcatcccttgctcacacct3' forward | NM_003181 |
| | | 5'acattctagggggcagagca3' reverse | |
| NES | 318 | 5'agcgttggaacagaggttg3' forward | NM_006617 |
| | | 5'tgggagcaaagatccaagac3' reverse | |
| MAGEA2 | 545 | 5'gggacaggctgacaagtagg3' forward | NM_005361 |
| | | 5'tctgaggacactctccagca3' reverse | NM_175742 |
| | | | NM_175743 |
| MAGEA3,6 | 399 | 5'gtaggaaggtggccgagttg3' forward | NM_005362 |
| | | 5'tgggatcccccaagatactg3' reverse | NM_005363 |
| | | | NM_175868 |
| MAGEA4 | 383 | 5'tcagggagcctctgccttac3' forward | NM_001011548 |
| | | 5'tattacccagcaggccatca3' reverse | NM_002362 |
| | | | NM_001011549 |
| | | | NM_001011550 |

| | | | |
|---|---|---|---|
| MAGEA8 | 319 | 5'tccagcagcaatgaagagga3' forward<br>5'gcaggccatcataggagag3' reverse | NM_005364 |
| MAGEB2 | 222 | 5'aggacccgagcgagtgtag3' forward<br>5'ccccagaaacagaagaggaa3' reverse | NM_002364 |
| MAGED1 | 247 | 5'cctccgttcctaccatgaga3' forward<br>5'ccaggtcagcagctcaaact3' reverse | NM_001005333<br>NM_006986<br>NM_001005332 |
| MAGED2 | 561 | 5'aaaggccacagaggtctcaa3' forward<br>5'cgggctttaggtgatctcag3' reverse | NM_014599<br>NM_177433<br>NM_201222 |
| GAGE1,2A,2B,<br>2C,2D,2E,10,12<br>C,12D,12E,12G,<br>12H,12I,12J,13 | 238 | 5'ctgagattcatctgtgtgaaatatga3' forward<br>5'aggcttcggcccttga3' reverse | NM_001468<br>NM_001040663<br>NM_001127212<br>NM_001098411<br>NM_001472<br>NM_001098407<br>NM_001127200<br>NM_001098413<br>NM_001098408<br>NM_001127199<br>NM_001098418<br>NM_001098409<br>NM_001098410<br>NM_001477<br>NM_001098406<br>NM_001098412 |

| | | | |
|---|---|---|---|
| RPL19 | 326 | 5'agggtacagccaatgcccga3' forward<br>5'ccttggataaagtcttgatgatc3' reverse | NM_000981 |

**Table S2:** RT-PCR primers for mouse genes studied

| Gene | Product (bp) | Primer sequences | Accession no. |
|---|---|---|---|
| Oct-4/Pou5f1 | 621 | 5'tggagactttgcagcctgag3' forward | NM_013633 |
| | | 5'catactcttctcgttgggatta3' reverse | |
| Nanog | 702 | 5'caagcggtggcagaaaaac3' forward | NM_028016 |
| | | 5'tggataagagcacccgactg3' reverse | |
| Magea4 | 224 | 5'tcggagccaaagggagttag3' forward | NM_020280 |
| | | 5'tgcacagtgggtcttcctgt3' reverse | |
| Magea1,2,3,5,6,8 | 361 | 5'actggcctccatccctgata3' forward | NM_020015 |
| | | 5'cttgggcataccctggacat3' reverse | NM_020016 |
| | | | NM_020017 |
| | | | NM_020018 |
| | | | NM_020019 |
| | | | NM_020020 |
| Mageb3 | 125 | 5'ttcaaatgggttttcacacacg3' forward | NM_008545 |
| | | 5'aattttatggttggtggggtca3' reverse | |
| Mageb1,2,3 | 435 | 5'gttccagtagttcagcccactgc3' forward | NM_010759 |
| | | 5'agtgctgggatcaatttccttc3' reverse | NM_031171 |
| | | | NM_008545 |
| Maged1 | 509 | 5'aaggggccaaatgattactctcag3' forward | NM_019791 |
| | | 5'tgatccccactgttgttctctt3' reverse | |
| Maged2 | 309 | 5'cagtgaccagagtcaggcttc3' forward | NM_030700 |
| | | 5'gagcgtctgatgggaatcttt3' reverse | |
| Hprt | 249 | 5'gctggtgaaaaggacctct3' forward | NM_013556 |
| | | 5'cacaggactagaacacctgc3' reverse | |
